# Near‐Sensor Reservoir Computing for Gait Recognition via a Multi‐Gate Electrolyte‐Gated Transistor

**DOI:** 10.1002/advs.202300471

**Published:** 2023-03-22

**Authors:** Xuerong Liu, Cui Sun, Zhecheng Guo, Xiangling Xia, Qian Jiang, Xiaoyu Ye, Jie Shang, Yuejun Zhang, Xiaojian Zhu, Run‐Wei Li

**Affiliations:** ^1^ CAS Key Laboratory of Magnetic Materials and Devices and Zhejiang Province Key Laboratory of Magnetic Materials and Application Technology Ningbo Institute of Materials Technology and Engineering Chinese Academy of Sciences Ningbo 315201 China; ^2^ Zhejiang Province Key Laboratory of Magnetic Materials and Application Technology Ningbo Institute of Materials Technology and Engineering Chinese Academy of Sciences Ningbo 315201 China; ^3^ Faculty of Electrical Engineering and Computer Science Ningbo University Ningbo 315211 China; ^4^ College of Materials Sciences and Opto‐Electronic Technology University of Chinese Academy of Sciences Beijing 100049 China

**Keywords:** gait recognition, multi‐gate electrolyte‐gated transistor, near‐sensor computing, reservoir computing, smart wearable electronics

## Abstract

The recent emergence of various smart wearable electronics has furnished the rapid development of human–computer interaction, medical health monitoring technologies, etc. Unfortunately, processing redundant motion and physiological data acquired by multiple wearable sensors using conventional off‐site digital computers typically result in serious latency and energy consumption problems. In this work, a multi‐gate electrolyte‐gated transistor (EGT)‐based reservoir device for efficient multi‐channel near‐sensor computing is reported. The EGT, exhibiting rich short‐term dynamics under voltage modulation, can implement nonlinear parallel integration of the time‐series signals thus extracting the temporal features such as the synchronization state and collective frequency in the inputs. The flexible EGT integrated with pressure sensors can perform on‐site gait information analysis, enabling the identification of motion behaviors and Parkinson's disease. This near‐sensor reservoir computing system offers a new route for rapid analysis of the motion and physiological signals with significantly improved efficiency and will lead to robust smart flexible wearable electronics.

## Graphical abstract

This work reports a multi‐gate electrolyte‐gated transitior (EGT)‐based reservoir device capable of parallel integrating and processing multi‐channel streaming signals. This reservoir can be integrated with multiple pressure sensors to extract the temporal features including the synchronization state and collective frequency in the sensory inputs. Accurate identification of gait patterns during bipedal movement is realized, providing new ideas for smart wearable electronics.

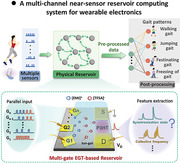

## 1 Introduction

**Figure 1:**
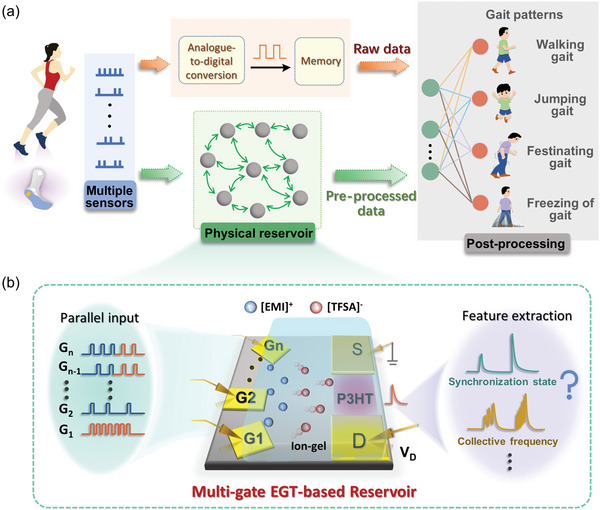
Near‐sensor reservoir computing for wearable electronics. a) Comparison between the digital computing and near‐sensor reservoir computing schemes for gait recognition. In the former case, redundant raw data is digitized, stored then transmitted for post‐processing. In the latter case, the data are pre‐processed by the physical reservoir for key feature extraction, which largely reduces the data amount for post‐processing, significantly improving the efficiency. b) Illustration of a flexible multi‐gate electrolyte‐gated transistor (EGT) as a reservoir for parallel signal processing, enabling the extraction of temporal features such as the synchronization state and collective frequency.

The efficient and accurate monitoring and analysis of the human physiological and motion signals are critical for disease diagnosis and health condition evaluation, which find widespread applications in human‐computer interaction, medical monitoring and sports healthy, etc.^[^ ^1^, ^2^, ^3^, ^4^ ^]^ For instance, the gait information, typically encoded in the bipedal movement produced multi‐channel time‐series pressure signals, can indicate the normal gaits (e.g., walking and jumping) and abnormal gaits (e.g., early‐stage and advanced‐stage Parkinson's disease).^[^ ^5^, ^6^, ^7^, ^8^ ^]^ As the key hardware that bridges the human body and external electronics, wearable electronics represented by the flexible strain sensors deployed on different body positions enable the acquisition of motion (gait, muscle, joint) and physiological (heart, brain wave) signals with a high spatiotemporal resolution and throughput.^[^ ^9^, ^10^, ^11^ ^]^ For data analysis, the acquired sensory signals must be digitized, stored, then transferred to an external digit computer or the cloud for processing.^[^ ^12^, ^13^, ^14^, ^15^ ^]^ Unfortunately, owing to the vast amount of data for processing, its transfer from the sensor nodes to the external processing units through limited bandwidth greatly limits data processing speed, and leads to severe energy consumption and security issues (**Figure** **1**a).^[^ ^16^, ^17^, ^18^, ^19^ ^]^

Near‐sensor computing where the computational tasks are implemented near the sensor units offers an alternative solution to mitigate the redundant data movement issue.^[^ ^15^ ^]^ Specifically, the raw sensory data is pre‐processed near the sensory nodes and the extracted data features are transferred for further analysis. The much‐reduced data capacity enables real‐time, accurate, and low‐power consumption data processing.^[^ ^15^, ^20^, ^21^, ^22^, ^23^, ^24^ ^]^ Reservoir computing (RC) is a recurrent neural network‐based computational paradigm used for time‐series signal analysis.^[^ ^25^, ^26^ ^]^ With fading memory, the reservoir device can nonlinearly transform the input data and extract the temporal features utilizing its internal dynamics, then projected them to a high‐dimensional space for a trained readout function to implement high‐level processing, e.g., classification and prediction.^[^ ^25^, ^27^, ^28^ ^]^ The reservoir‐based signal processing is high speed, low cost, and energy efficient, therefore, serves as an ideal candidate to be integrated with the sensors for the implementation of near‐sensor computing paradigm.^[^ ^27^, ^29^, ^30^, ^31^ ^]^

Various emerging nanodevices represented by memristors have been utilized for physical reservoirs.^[^ ^29^, ^32^, ^33^ ^]^ By leveraging the internal ionic dynamics that offer nonlinearity and short‐term memory effects, these reservoir devices have been successfully used for different task implementations including hand‐written letter recognition, spoken‐digit prediction, neural spike analysis, language learning, breast tumors classification, etc.^[^ ^34^, ^35^, ^36^, ^37^, ^38^ ^]^ However, memristors with a two‐terminal structure typically respond to single‐channel inputs individually, which are not suited for scenarios requiring multi‐channel data integration and correlation feature extraction (e.g., gait recognition involving bipedal movements).^[^ ^39^, ^40^, ^41^, ^42^ ^]^ Besides, the memristors based on stochastic ion movements and local filaments formation/rupture usually suffer from variation issues that degrade device stability, and the rigid nature of the reported reservoir devices also prevents conformal integration with the flexible wearable sensors.^[^ ^43^ ^]^ Therefore, a compact reservoir device enabling multiple channel signal response, integration, feature extraction as well as good stability and mechanical flexibility is desirable.

In this work, we present a physical reservoir device capable of parallel integrating and processing multi‐channel streaming signals for near‐sensor computing. The reservoir is an organic multi‐gate electrolyte‐gated transistor (EGT) with inherent non‐linearity and short‐term memory characteristics offered by the stable ion movement. The device can respond to streaming voltage pulses transmitted by multi‐channels in parallel and implement nonlinear signal integration, allowing the temporal correlations between the inputs to be effectively extracted. The reservoir device with good mechanical flexibility can be integrated with flexible sensors, resulting in a flexible intelligent pressure perception system for on‐site monitoring and analysis of the gaits. Neural network simulation results demonstrate the identification of motion gestures and gait diseases with an accuracy of over 93%. This system capable of recognition of temporal and spatial features of input signals from multiple sensor units, provides a new idea for high‐performance flexible wearable systems.

## 2 Results and Discussion

### 2.1 Device Materials and Structure

The physical reservoir device is schematically illustrated in Figure 1b. It is an EGT prepared on a PET flexible substrate, using the ion‐gel made of ionic liquid [EMI][TFSA] and P(VDF‐HFP) as the electrolyte and organic semiconductor P3HT as the channel material. Au was deposited on a PET substrate by electron beam evaporation as the gate, source, and drain of the transistor device, and the width between the source and drain electrodes is 100 µm. Particularly, the device can be designed with a multiple in‐plane gate, which is equally distant (1400 µm) from the active region of the channel (Figure S2, Supporting Information). The multi‐channel electric inputs applied on these gates in parallel concurrently drive the migration of mobile ions in the electrolyte toward the channel to achieve dynamic conductance modulation as a result of ion doping. The integration and conversion of the electric inputs to the combined conductance changes are expected to extract the temporal features such as the synchronization state and collective frequency in the input data.

### 2.2 Short‐Term Memory Effect of the Multi‐Gate EGT‐Based Reservoir

**Figure 2:**
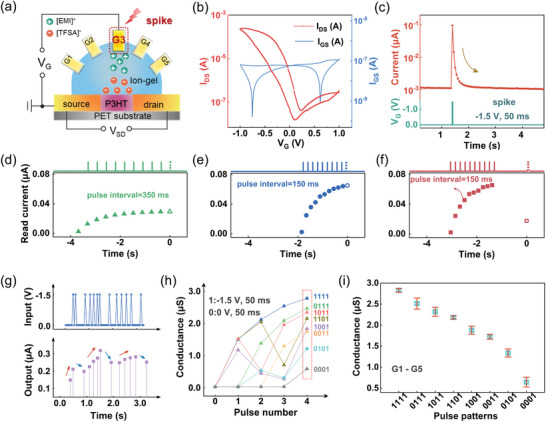
An EGT‐based reservoir device. a) Electrical setup for EGT measurement, where the electrical stimulation is applied on single gates, e.g., G3. b) Typical transfer curve of the EGT device measured during dual sweep from 1.0 V → −1.0 V → 1.0 V. c) Current evolution during single voltage pulse (−1.5 V, 50 ms) stimulation. d–f) Read current responses in the device subjected to ten voltage pulses (−1.5 V, 50 ms) with different pulse intervals. The device currents recorded at *t* = 0 ms (represented as open symbols) are affected by the pulses applied in the past. g) Read current response in the device stimulated by voltage pulses (−1.5 V, 50 ms) with varying pulse intervals. h) Evolution of the device conductance with the pulse strings having different pulse numbers and sequences. Different conductance states are reached after pulse excitation (marked in the red dashed box), which reflect the temporal feature in the input pulses. i) Plots of the device conductance with the pulse patterns in (h), averaged over five gates (G1–G5).

Electrical measurements were first carried out on the device with a single gate under electric stimulation (**Figure** **2**a). With the gate voltage dual sweeping from 1 to −1 V, and a *V*_read_ (*V*_DS_ = −0.8 V) voltage, the device exhibits a hysteresis in the *I*_DS_ –*V*_G_ loop at negative gate voltages, and the conductance change ratio reaches 10^5^, suggesting the pronounced conductance modulation effect. (Figure 2b) The device exhibits volatile conductance changes, where the channel conductance relaxes spontaneously upon programming. The mechanistic studies reveal anion accumulation at the channel interface that induces electrostatic regulation and conductance enhancement.^[^ ^40^, ^44^ ^]^ The electrical response of the EGT to single‐gate pulse stimuli was further measured. The spatiotemporal electrical input was applied on the gate of the transistor, and the current evolution with a single voltage pulse (−1.5 V, 50 ms) is shown in Figure 2c. During pulse stimulation, a quick rise of the read current from ≈1 to ≈100 nA was detected, which gradually decay back to ≈1 nA after ≈800 ms. This observation indicates the short‐term dynamics and fading memory effects, caused by the transient accumulation then back diffusion of the [TFSA]^−^ anions at the P3HT/ion‐gel interface that tunes the conductivity through electrostatic doping. Further studies show that the variations of the cycle‐to‐cycle performance for single gates, and the device‐to‐device performances for different gates are both less than 4% (Figure S5, Supporting Information). The high uniformity indicates good device stability enabled by the homogenous ion movements in the ionic electrolyte in comparison to the memristor‐based reservoir driven by the stochastic ion migration‐induced random formation/rupture of the filaments.

The effect of pulse trains consisting of single pulses at different intervals on the device behavior was studied. Figure 2d–f shows the evolution of the device read current on ten pulses (−1.5 V, 50 ms) with intervals of 350 and 150 ms respectively. It was found that pulse trains with smaller time intervals, i.e., higher frequencies, lead to a quicker elevation in the device current. This is ascribed to the spontaneous back diffusion of fewer ions at the reduced pulse intervals that enhance ion accumulation, thus promoting fast channel doping and conductance enhancement. Upon a pronounced accumulation in device current, the conductance state eventually relaxes to the low conductance state if a large time interval exists before the next pulse arrives (Figure 2f). For the distinct device responses in Figure 2d–f, one can see that the read current at *t* = 0 ms (marked with an open symbol in each plot) is largely influenced by the inputs that occurred in the near past (Figure 2d,e) rather the distant past (Figure 2f), owing to the short‐term memory dynamics, consistent with the echo state property of the reservoir.^[^ ^34^, ^37^ ^]^ The device can steadily and dynamically evolve with a pulse train composed of discrete single pulses (−1.2 V, 50 ms) having varying pulse intervals (50–350 ms), where the rise and decay behaviors of the read current clearly reflect the temporal sequence characters of the electric pulses (Figure 2g).

A good reservoir features good separability that can differentiate different time‐series inputs based on the reservoir states. The separability of this EGT is tested by pulse streams with different temporal features. In one such test, the pulse stream consists of electrical pulses (−1.5 or 0 V, 50 ms width, 100 ms interval), with different numbers and sequences, namely, 0001, 0011, 0101, 1001, 0111, 1011, 1101, and 1111 are applied, where “1/0” corresponds to a pulse with an amplitude of −1.5/0 V, respectively. As shown in Figure 2h, upon the electric stimuli, the pulse strings with more pulse number and later firing timing, leads to a higher conductance state (dashed box). More important, these conductance states corresponding to the reservoir states, can be clearly separated, demonstrating good separability of the EGT‐based reservoir. Similar tests were performed on five device gates, distinguishable margins among the averaged conductance states with small variations (≈7.1%) measured from five devices were obtained, again verifying the excellent stability (Figure 2i).

### 2.3 Multi‐Channel Signal Integration and Processing

**Figure 3:**
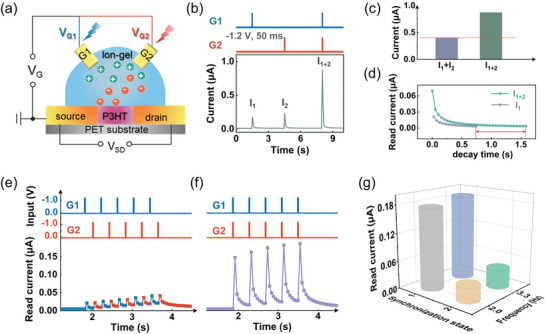
EGT‐based reservoir for dual‐channel signal processing. a) Electrical setup for EGT measurement, where the electrical stimulation is applied on dual gates, e.g., G1 and G2. b) Device current triggered by voltage spikes (−1.2 V, 50 ms) applied on G1 and G2 at different moments. c) Comparison of the summed current for the electric pulses applied on the gates separately (*I*_1_+*I*_2_) and simultaneously (*I*_1+2_), extracted from (b). d) Relaxation characteristics of the device current upon pulse programming, extracted from (b). e,f) Evolution of the device current with the pulse trains containing five pulses (−1.2 V, 50 ms width, 350 ms interval) applied on G1 and G2 asynchronously (e) and synchronously (f). g) Dependence of the device current on the synchronization state and collective frequency in the inputs. Synchronization state 1: synchronously; synchronization state 2: unsynchronously

To further examine the potential of the EGT‐based reservoir for multi‐channel signal integration, the dynamics of the EGT‐based reservoir in response to two individual pulse trains were studied. Experimentally, the two pulse trains were separately applied to the two gates of the EGT as inputs, and the channel read currents were recorded as outputs (**Figure** **3**a). In Figure 3b, a single spike at controlled timing steps was applied to each of the two gates. While the individual spike (−1.2 V, 50 ms) separately applied either on G1 or G2 produces transient current enhancement with comparable intensity, the paired spikes simultaneously applied on the dual gates excited the device to a much higher conductance state. Careful calculation of the changed current intensity indicates a supralinear integration of the device current, i.e., *I*_1+2_ > *I*_1_+*I*_2_, where *I*_1_ and *I*_2_ denote the current excited by G1 and G2 alone respectively, and I_1+2_ denotes the current triggered by G1 and G2 simultaneously (Figure 3c). Moreover, the retention test implies that the current relaxed from the high conductance state to the resting state takes ≈1650 ms, much longer than that underwent single channel stimulation (≈800 ms; Figure 3d). The enhanced current modulation and memory retention are a result of the strengthened ion accumulation and extended ion diffusion process, contributed by the parallel and concurrent driving of the [TFSA]^−^ anions towards the P3HT channel. The exhibition of distinct device dynamics to dual‐channel signals having different temporal correlations open up the possibility of extracting the temporal features in the inputs using the EGT device.

The evolution behaviors of device current in response to dual pulse trains having more complex temporal features were studied. Figure 3e,f shows the unsynchronized and synchronized dual‐channel pulse trains (−1.2 V, 50 ms width, 350 ms interval) applied on the dual gates in parallel and the corresponding device responses. For the unsynchronized case, the read current was excited to a higher level by each pulse alternatively applied on the dual gates, and the overall conductance decays during the pulse intervals. Notably, if the interval between the pulse applied on G1 and G2 is within the relaxation window, the residual current enhancement induced by the previous pulse can be cumulated to the following pulse stimulation, giving rise to the progressive increment in device current. Compared to the unsynchronized case, the synchronous inputs generally lead to much enhanced responses, owing to the strong accumulation and slow relaxation of ions at the electrolyte/channel interface. We also studied the pulse frequency‐dependent integration behaviors of the EGT‐based reservoir. Remarkably, for both synchronized and unsynchronized cases, the higher the collective frequency, the larger the conductance is enhanced. Figure 3g plots the read currents as a function of the pulse number at different collective frequencies (e.g., 2.0 and 3.3 Hz) for the unsynchronized and synchronized states. The evolution rate and strength of the device current naturally encode the synchronization and collective frequency characteristics in the two streaming inputs.

### 2.4 Gait Recognition

**Figure 4:**
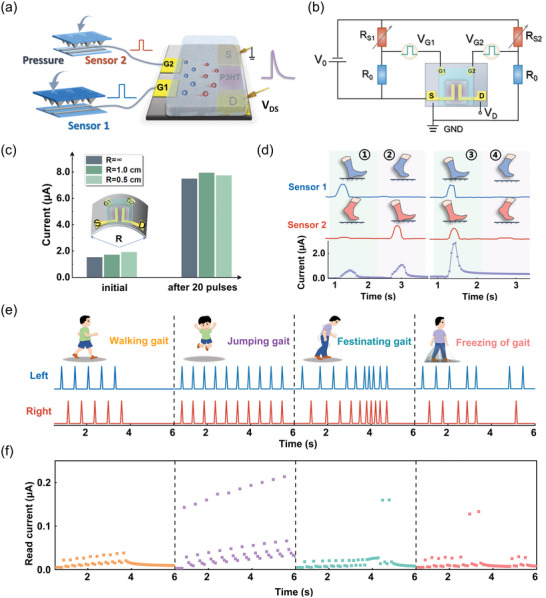
EGT‐based pressure sensory system for gait analysis. a) Schematic illustrating the connection of two pressure sensors with an EGT‐based reservoir. b) Circuit diagram of the EGT‐based pressure sensory system, comprising the EGT, pressure sensors (*R*_S1_ and *R*_S2_), voltage sources (*V*_0_ = 3.3 V), and serial resistors (*R*_0_ = 4 kΩ). c) Effect of the bend radius on the conductance change behavior in the device under pulse stimulation. Inset: Schematic showing a curved EGT. d) Bipedal movement produced voltage pulses through the pressure sensors and the induced device current changes. e,f) Four typical gait patterns, namely, “Walking Gait”, “Jumping Gait”, “Festinating Gait” and “Freezing of Gait” (e), and the corresponding device responses (f).

To examine the feasibility of using the multi‐gate EGT‐based reservoir for intelligent wearable electronics, we integrate the reservoir device with flexible wearable sensors (**Figure** **4**a). Specifically, two flexible resistive pressure sensors whose resistance decreases with the applied pressure were connected with the EGT in serial through voltage divider circuits (Figure 4b). The pressures loaded on the sensors serve as the inputs to reduce the sensor resistances and increase the effective voltage dropped across the EGT device, resulting in the enhancement of the reservoir conductance and generation of reservoir states. The reservoir device consisted of the organic channel layer (P3HT) and ion‐gel ([EMI][TFSA]‐P(VDF‐HFP)) with intrinsic flexibility allowing for stable operation under strain, implied by Figure 4c showing the weak dependence of the current changes on the bending radium (*R* = ∞, 0.5, and 1.0 cm). Thus, the pressure sensors along with the reservoir device can be deployed on the plantar to collect and process the gait data during bipedal motion. To be compatible with the task scene and achieve the best device performance, the foot‐ground contact duration was optimized to be ≈50 ms and the time interval was defined to be within 300 ms. The readout current was sampled every 100 ms. Figure 4d shows the real‐time generation of voltage pulses by foot movement through the sensors (left foot, sensor 1; right foot, sensor 2), and the corresponding device responses. Interestingly, the transient contact of the pressure sensor with the ground produces pulsed voltage signals, and the inputs for the EGT are essentially dual‐channel pulse‐like streaming data, which can effectively drive the device.

Four typical gait patterns were chosen for the evaluation, namely, “Walking gait”, “Jumping gait”, “Festinating gait (FSG)”, and “Freezing of gait (FOG)”, where both FSG and FOG are two typical gait patterns of Parkinson's disease, occurring in the early‐stage and advanced‐stage, respectively.^[^ ^6^, ^7^, ^45^ ^]^ Specifically, the “Walking gait” corresponds to a periodic motion by moving the left and right feet alternately (Figure 4d, process 1,2). The “Jumping gait” is characterized by both feet leaving from or landing on the ground at the same time (Figure 4d, process 3,4), at a lower frequency than the walking mode. The distinctive feature of the abnormal “FSG” is that the patient walks faster over time, accompanied by an occasional landing on both feet. The “FOG” is characterized by irregular foot movement involving alternating or landing at the same time, and is distinguished by the fact that the patient is frozen in the process of walking and resumes walking after a transient stop. Figure 4e shows the four typical gait patterns fed to the transistors, collected from the sensors during bipedal motion (Figure S11, Supporting Information), and the measured device responses are presented in Figure 4f. The duration of the inputs is ≈6 s, creating 60 conductance states representing reservoir states under a sampling rate of 100 ms. The walking and jumping gaits were found to produce reservoir states similar to those in Figure 3e,f. For the FSG, the device current behaviors like that in the walking mode at the beginning, then showed a trend of enhancement due to the acceleration in the walking speed. The occasional landing of both feet that drives a transient and significant conductance enhancement was captured. The test result for the FOG is much more disordered, involving the random occurrences of the fast/slow and rise/decay of the device current over time. These results indicate that the reservoir device can effectively respond to the sensory inputs and produce reservoir states encoding the key gait features with much‐reduced data amount for further analysis.

**Figure 5:**
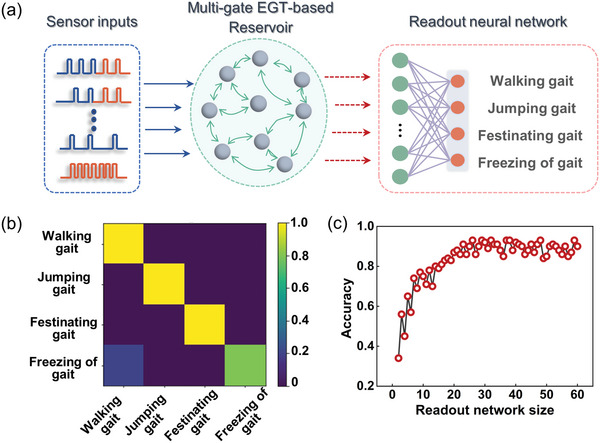
Gait identification using the near‐sensor RC system. a) Schematic of the near‐sensor RC system, containing the pressure sensors, the EGT‐based reservoir, and the readout neural network. b) False color confusion map showing the simulated identification results. The occurrence probability for each gait pattern is represented by the colors in the color scale. c) Recognition accuracy as a function of readout network size.

The reservoir states produced by the EGT encoding the gait feature were further analyzed by a readout layer made of a 60 × 4 fully connected neural network (**Figure** **5**a), where the recorded 60 reservoir states were used as the inputs and the four output neurons corresponded to the gait types for classification. The dynamics of the reservoir can be well modeled and predicted by using the fitted conductance change parameters extracted from the device during and after pulse stimulation (Figure S13, Supporting Information), we simulated 1000 sets of samples for the four gait types based on the device parameters with added variations in pulse durations and intervals. 500 of the datasets were selected for readout layer training and the rest for inference. After repeated training using the logistic regression approach with the goal to minimize the output error, the features of the reservoir states were successfully learned by the network. Figure 5b shows the classification results during inference, where the color confusion matrix plots the identified type as a function of the ground truth result and the pixel darkness represents the accuracy of the corresponding results. This system gives an accuracy of 93% for gait recognition. We further studied the effect of the readout layer size on the recognition accuracy. It shows that reducing the size of the network results in a decrease in the recognition accuracy, however, an accuracy of ∼90% can still be obtained at a network size of 30 (Figure 5c). Thus, a trade‐off between the costs of training the network and the accuracy is required to achieve considerable system performance.

The parallel integration of the pressure signals during gait motion and extracting their temporal correlations near the sensor nodes enabled by the reservoir device greatly reduces the amount of data for neural network analysis and computational costs. Along with the advantages in terms of reduced energy consumption, processing latency, and cost of the readout layer training, the simple device structure and good mechanical flexibility further facilitate the integration with various flexible sensors. While the proof‐of‐concept reservoir device was used for simple biped gait studies in this work, more complex multi‐channel data processing is achievable by leveraging an increased number of gate terminals. Besides, careful engineering of the electrode distribution, ionic species in the electrolyte and programming conditions are expected to create richer device dynamics, opening up opportunities for the reservoir‐based near‐sensor computing system to analyze more complex data such as the electrocardiogram (ECG) based electrophysiological signals. Moreover, recent studies show that the EGTs can be used as artificial synapses for learning and memory mimicry, and further integrated with sensors and actuators for artificial sensorimotor nerve emulation.^[^ ^46^, ^47^, ^48^, ^49^, ^50^ ^]^ These encouraging progresses promise the bright future of EGTs towards high‐performance artificial intelligence systems that find wide applications in neuroprosthesis, humanoid robotics, and biohybrid electronics for human‐machine interfaces.

## 3 Conclusion

In summary, we developed a flexible, multi‐gate EGT‐based reservoir for near‐sensor computing in wearable electronics. The reservoir device integrates the functionalities of multi‐channel inputs integration and temporal features extraction, with good stability and low power consumption. Such reservoir devices can preprocess the gait signals during human motion near the sensor ends, and enable a trained neural network with a reduced size to recognize the gait patterns with high accuracy. Our work provides new solutions for building smart wearable electronic systems with powerful on‐site and real‐time sensing and processing capabilities.

## 4 Experimental Section

### Materials

The semiconductor poly (3‐hexylthiophene‐2,5‐diyl (stereoregular) (P3HT) and dichlorobenzene (98%, water < 50 ppm) were purchased from Aladdin Company. The polymer poly(vinylidene fluoride‐co‐hexafluoropropylene) (P(VDF‐HFP)) was obtained from Macklin and the ionic liquid 1‐ethyl‐3‐methylimidazoliumbis (trifluoromethylsulfonyl) amide ([EMI][TFSA]) was sourced from Annaiji company.

### Preparation of P3HT Solution and Ion‐Gel Film

The solution was obtained by dissolving P3HT powder in dichlorobenzene solution at a concentration ratio of 10 mg mL^−1^, and vigorous stirring at 50 °C for 2 h to fully dissolve P3HT in dichlorobenzene solution. P(VDF‐HFP) and [EMI][TFSA] were dissolved in acetone with a weight ratio of 1:4:7 under vigorous stirring at 50 °C for 4 h, and then spin‐coated on a glass substrate to obtain an ion‐gel film.

### Fabrication of the Multi‐Gate EGT

The fabrication process of the device is described as follows. The PET substrate was placed in acetone, alcohol, and deionized water for ultrasonic cleaning. Electron beam evaporation of Au electrodes was performed with a micrometer‐sized metal shadow mask to obtain the source, drain, and in‐plane gates. The channel had a length of 1000 µm and a width of 100 µm, the P3HT solution was spin‐coated to cover the channel, cured at 70 °C for 2 h to obtain a P3HT film (≈55 nm) above the channel. Finally, the ion‐gel film (≈18.2 µm) was transferred to complete the device fabrication.

### Characterization

Electrical properties of the devices were conducted on a Lake‐shore probe station with a Keithley 4200 semiconductor parameter analyzer in the atmosphere and at room temperature. During device operation, the voltage spikes as the input signals were applied on the gate electrodes, and a constant bias voltage (0.1 V) as the read voltage was applied between the source‐drain electrodes. During the application of voltage spikes, we collected I_DS_ as the output current 100 ms after the application of each voltage spike.

### Simulation

For the gait pattern recognition, the readout layer is a 60 × 4 fully connected neural network with four output neurons corresponding to the “Walking”, “Jumping”, “FSG” and “FOG” gait types. For training, 1000 sets of samples were simulated for the four gait types based on the device parameters with added variations in pulse durations and intervals. Five hundred of the datasets were selected for readout layer training and the rest for inference. A linear regression calculation was used to calculate the output weights based on the reservoir state and to minimize the error between the output results and the target results. After training, the data for the test were analyzed by the trained network to evaluate the recognition accuracy.

## Conflict of Interest

The authors declare no conflict of interest.

## Supporting information

Supporting Information

## Data Availability

The data that support the findings of this study are available from the corresponding author upon reasonable request.
