# Supplementary material for: Near‐Sensor Reservoir Computing for Gait Recognition via a Multi‐Gate Electrolyte‐Gated Transistor
Source: Adv Sci (Weinh). 2023 Mar 22;10(15):2300471. doi: 10.1002/advs.202300471 (PMC10214258; doi:10.1002/advs.202300471)
Supplement: Supplementary file 1 — Supporting Information [file ADVS-10-2300471-s001.pdf]

Supporting Information

Near-sensor Reservoir Computing for Gait Recognition via a Multi-gate Electrolyte-gated Transistor

Xuerong Liu [a, b]‡, *Cui Sun* [a, b]‡, *Zhecheng Guo* [c], *Xiangling Xia* [a, b], *Qian Jiang* [a, b, d], *Xiaoyu Ye* [a, b, d], *Jie Shang* [a, b], *Yuejun Zhang* [c], *Xiaojian Zhu* [a, b]*, *Run-Wei Li* [a, b]*

X. Liu, C. Sun, X. Xia, Q. Jiang, X. Ye, Prof. J. Shang, Prof. X. Zhu, Prof. R.-W. Li
[a] CAS Key Laboratory of Magnetic Materials and Devices, and Zhejiang Province Key Laboratory of Magnetic Materials and Application Technology, Ningbo Institute of Materials Technology and Engineering, Chinese Academy of Sciences, Ningbo 315201, China
[b] Zhejiang Province Key Laboratory of Magnetic Materials and Application Technology, Ningbo Institute of Materials Technology and Engineering, Chinese Academy of Sciences, Ningbo 315201, China
* Corresponding author: zhuxj@nimte.ac.cn, runweili@nimte.ac.cn

Z. Guo, Prof. Y. Zhang
[c] Faculty of Electrical Engineering and Computer Science, Ningbo University, Ningbo 315211, China

Q. Jiang, X. Ye
[d] College of Materials Sciences and Opto-Electronic Technology, University of Chinese Academy of Sciences, Beijing 100049, China

‡These authors contributed equally to this work.

Supplementary Figures

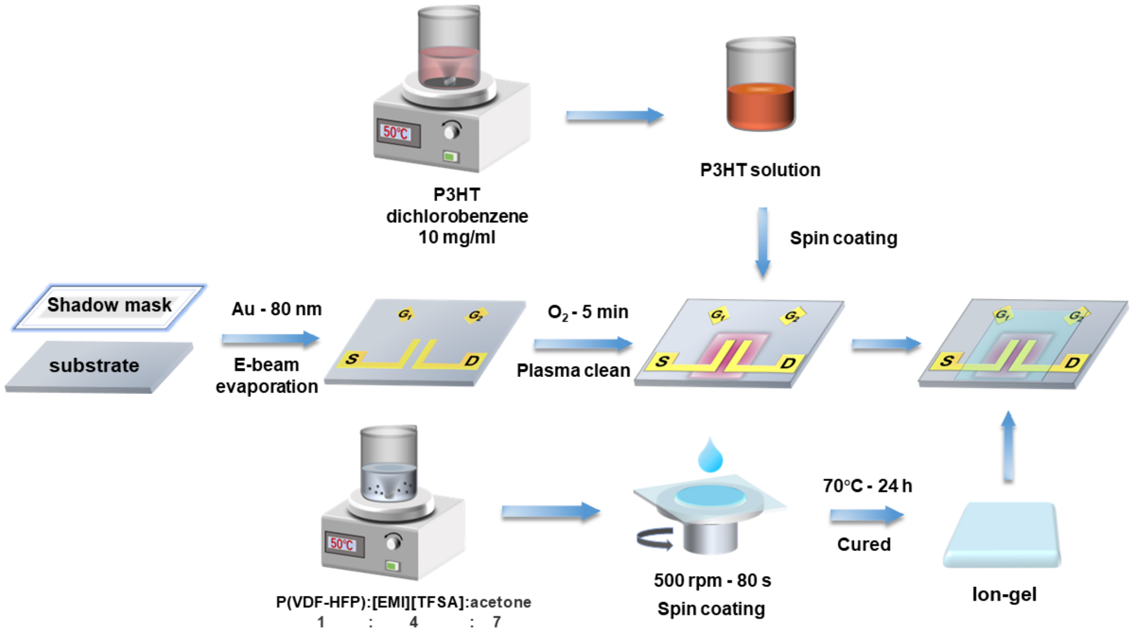

Figure S1. The flowchart showing the fabrication process of the EGT device.

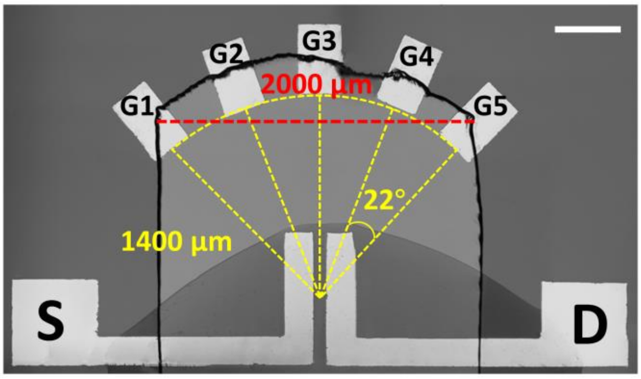

Figure S2. Optical image of the P3HT-based multi-gate EGT. For gait encoding, G1 and G5 were used. Scale bar: 500 μm.

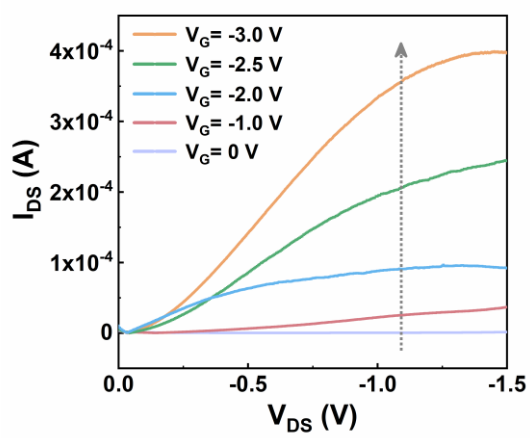

Figure S3. Typical output curves (I_{DS}-V_{DS}) of the EGT device at different gate voltages.

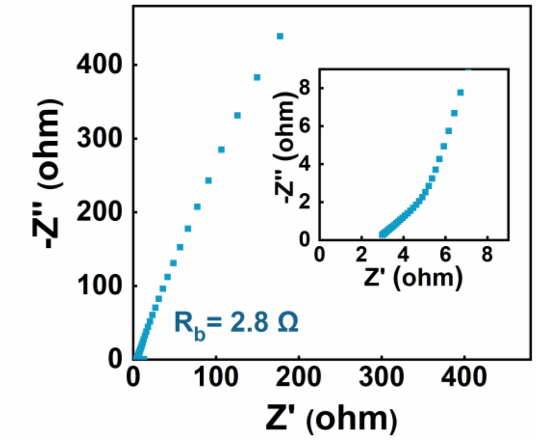

Figure S4. Nyquist plot showing the AC impendence performance of the P(VDF-HFP) ion-gel.

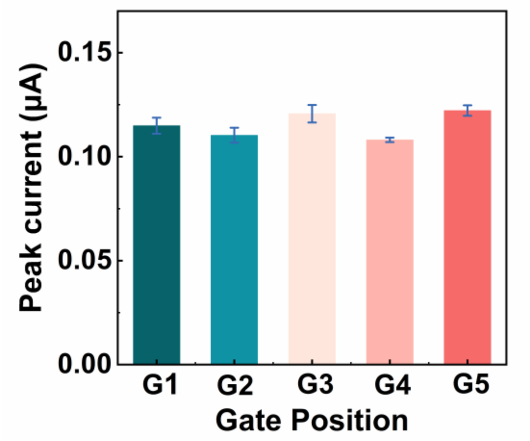

Figure S5. Peak current distribution for different gates under single pulse (-1.5 V, 50 ms) stimulation.

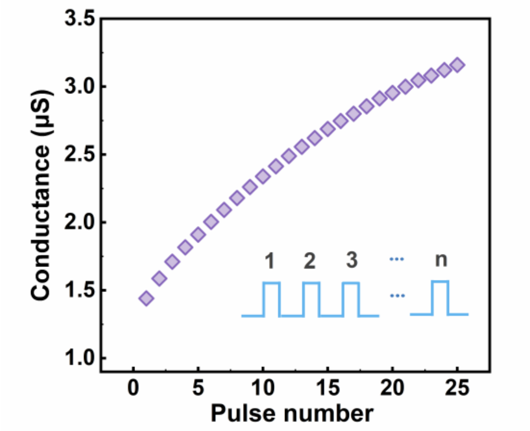

Figure S6. Device conductance as a function of the number of applied electrical pulses (-1.2 V, 50 ms width, 100 ms interval), showing nonlinear conductance changes.

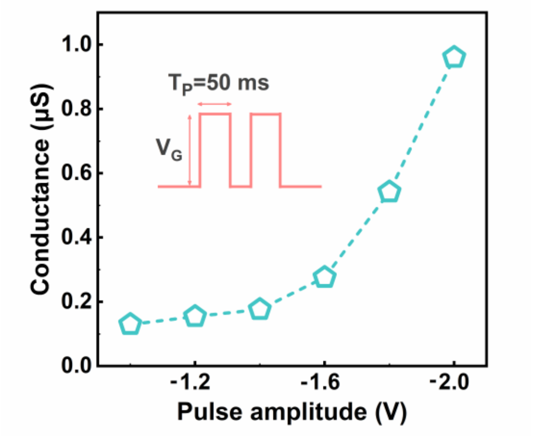

Figure S7. Device conductance as a function of the amplitude of the applied electric pulses (-1.0 - -2.0 V, 50 ms), showing nonlinear conductance changes.

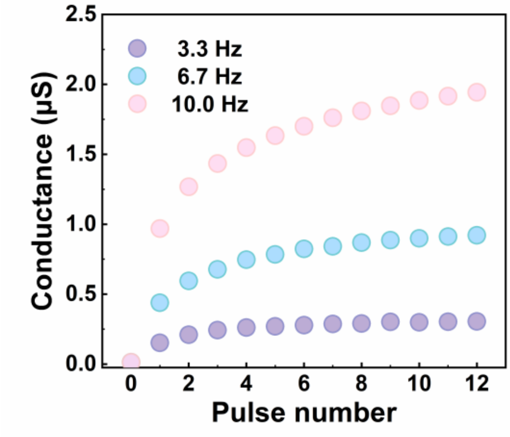

Figure S8. Evolution of the device current with the number of the pulses (-1.2 V, 50 ms) at different frequencies.

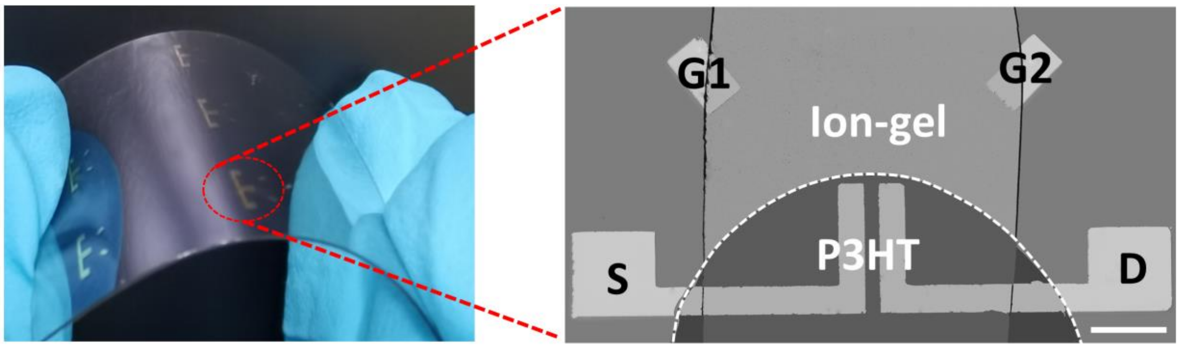

Figure S9. Optical image of the dual-gate EGT device deposited on a PET substrate under strain. Scale bar: 500 μm.

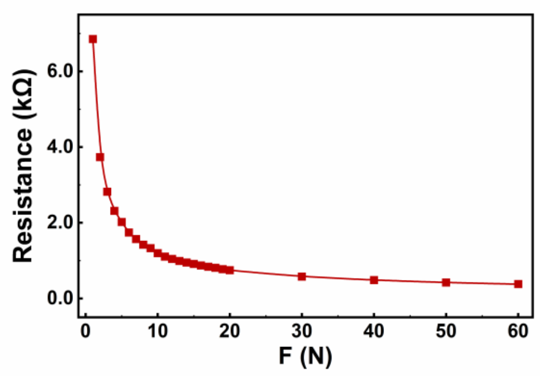

Figure S10. Electrical resistance of the pressure sensor as a function of the applied pressure amplitude.

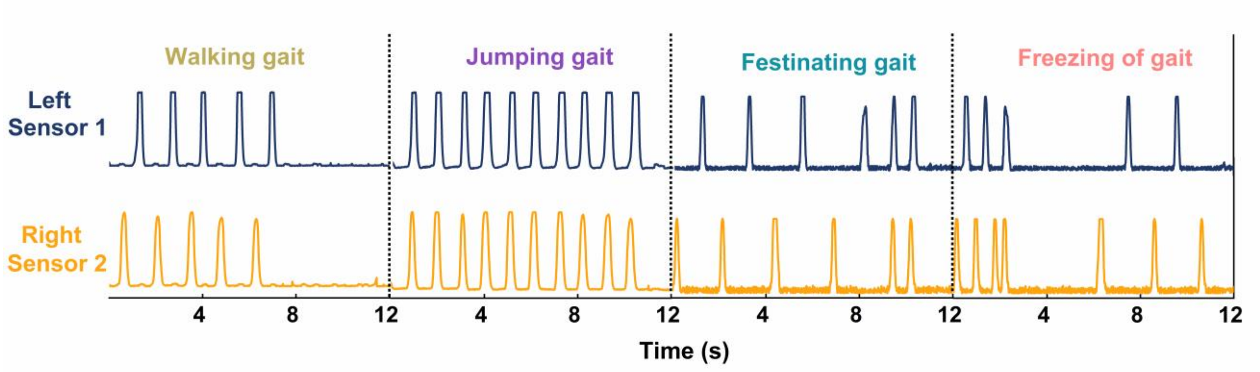

Figure S11. Voltage pulses produced by the pressure sensors corresponding to different gait patterns.

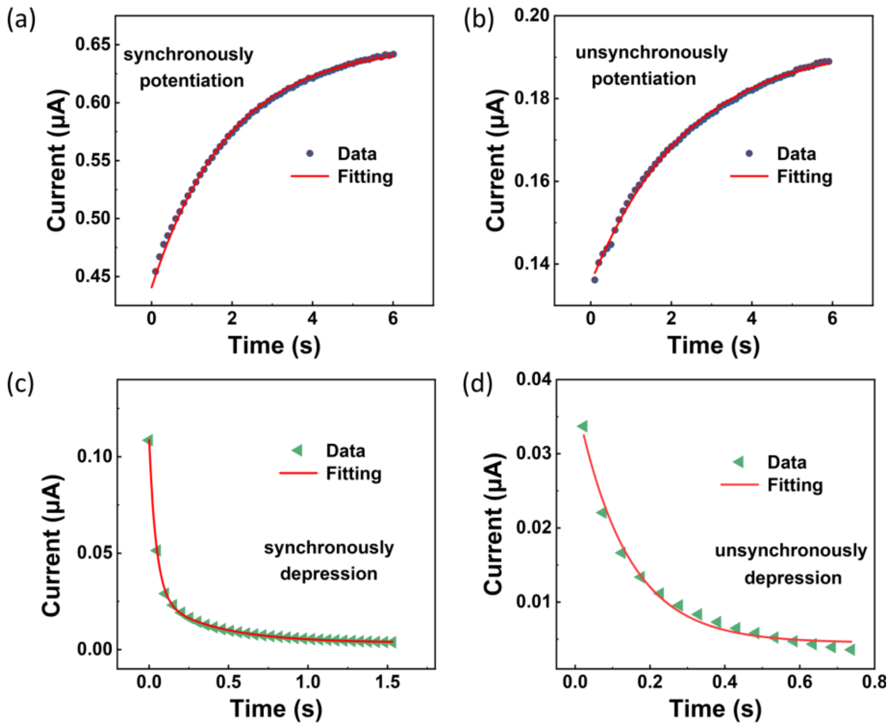

Figure S12. (a-b) Potentiation characteristics of the EGT device in response to (a) synchronous and (b) unsynchronous pulse inputs. (c-d) Depression characteristics of the EGT device after (c) synchronous and (d) unsynchronous pulse inputs.

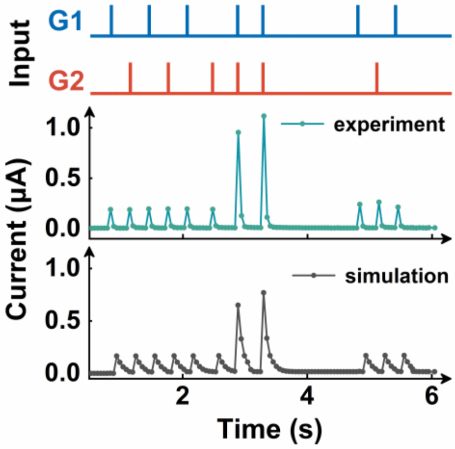

Figure S13. Read current evolution in the device excited by two voltage pulse trains with varied synchornization states. The experimental results are in good agreement with the simulation results based on the dynamics of the transistor.

Supplementary Table

Table S1. Parameters used in simulation for the potentiation and depression processes.

Parameter	Value (a)	Value (b)	Value (c)	Value (d)
τ	2.262	1.472	0.015	0.083
I_0	199.199	59.152	303.421	1.351
β	1	1	0.9	0.9
C_0	655.487	194.655	0.771	1.088

Supplementary Note1

The AC conductivity of the P(VDF-HFP)/[EMI][TFSA] ion-gel can be calculated by the Eq. (1):

$$\sigma = \frac{L}{S \times R_b} \qquad (1)$$

where L denotes the thickness of the ion-gel, S denotes the area of contact between the ionic gel and the electrode and R_b is the interfacial impedance of the ion-gel (**Figure S4**). The calculated result show that the conductivity of the ion-gel is ~1.78 mS/cm

Supplementary Note2

The ion dynamics of multi-gate EGT is caused by the gate voltage driving the emigration of $[TFSA]^-$ ions in the ion gel. ions accumulate at the P3HT/electrolyte interface, increasing the conductance of the device, and after the applied gate voltage is removed, the accumulated ions spontaneously diffuse, leading to a decrease in ion concentration and a decrease in device conductance.

Figure S12 shows the potentiation and depression behaviors of the EGT device in response to the synchronized and unsynchronized voltage inputs. During potentiation, the current evolution behaviors (**Figure S12a-b**) can be fitted by using a stretched exponential function shown in Eq. (2).

$$I = -I_0 \left(e^{-\frac{t}{\tau}}\right)^{\beta} + C_0 \qquad (2)$$

where I_0, τ, β denote the prefactor, characteristic decay time constant and the stretch index, respectively. The characteristic decay time constant (τ) was calculated to be 2.262 s and 1.472 s for the synchronous and unsynchronous state.

For the depression process, we used the stretched exponential function as shown in Eq. (3) for the fitting (**Figure S12c-d**),

$$I = I_0 \left(e^{-\frac{t}{\tau}}\right)^{\beta} + C_0 \qquad (3)$$

where the characteristic decay time constant (τ) of 0.015 s for the synchronous state and 0.083 s for the unsynchronous state can be obtained. The detailed parameters for device fitting are shown in **Table S1**.

Supplementary References

[1] C. Du, F. Cai, M. A. Zidan, W. Ma, S. H. Lee, W. D. Lu, *Nat. Commun.* **2017**, *8*, 2204.

[2] X. Zhu, Q. Wang, W. D. Lu, *Nat. Commun.* **2020**, *11*, 2439.

[3] C. Sun, X. Liu, Q. Jiang, X. Ye, X. Zhu, R. W. Li, *Sci. Technol. Adv. Mat.* **2023**, *24*, 2162325.

[4] K. H. Lee, M. S. Kang, S. Zhang, Y. Gu, T. P. Lodge,C. D. Frisbie, *Adv. Mater.* **2012**, *24*, 4457.
